# Blood pressure status, JSH 2019-based control rate, and associated factors among community-dwelling adults: The NOSE study

**DOI:** 10.1038/s41440-026-02622-8

**Published:** 2026-04-06

**Authors:** Phouvanh Chanthavong, Keigo Kobayashi, Yuya Akagi, Hiroko Yoshida, Michiko Kido, Kayo Godai, Yuka Fukata, Yuka Tachibana, Saya Terada, Liyu Shi, Chihiro Anzai, Yurie Maeyama, Haruna Kikuchi, Yuka Yokoyama, Arisa Wada, Makiko Higashi, Takeshi Kikuchi, Fumie Matsuno, Sho Nagayoshi, Kei Asayama, Takayoshi Ohkubo, Hiromi Rakugi, Yasuharu Tabara, Mai Kabayama, Kei Kamide

**Affiliations:** 1 Graduate School of Medicine, The University of Osaka, Osaka, Japan; 2 Graduate School of Nursing, Osaka Metropolitan University, Osaka, Japan; 3 Nose Town, Osaka, Japan; 4 OMRON HEALTHCARE Co., Ltd, Kyoto, Japan; 5 Department of Hygiene and Public Health, Teikyo University School of Medicine, Tokyo, Japan; 6 Graduate School of Public Health, Shizuoka Graduate University of Public Health, Shizuoka, Japan

**Keywords:** Home blood pressure, Hypertension guidelines, Community-Dwelling Adults, Achievement rate of blood pressure target

## Abstract

The Japanese Society of Hypertension Guidelines (JSH 2019) introduce stricter blood pressure (BP) targets, but BP status (hypertension prevalence and treatment) and control rates under these criteria, particularly by home BP monitoring, remain limited. This study investigated BP status, guideline-based BP control rates, and associated factors in a community-dwelling population. We analyzed baseline data (2020–2021) from 623 participants (mean age 67.6 years; 37.4% men) in the NOSE Study. BP status and control were defined using JSH 2019 criteria, with thresholds modified by age and comorbidities and 5 mmHg lower for home BP. Office BP and 30-day mean morning and evening home BP values (≥14 days) were assessed. Multivariable regression analysis was used to identify factors associated with hypertension prevalence, treatment status, and poor BP control. Hypertension prevalence was 66.8% and was associated with older age and higher body mass index. Approximately half of adults with hypertension were untreated, despite having higher BP levels, and tended to be younger with fewer comorbidities. Among treated participants, BP control rates were 22.9% based on office BP and 7.3% based on morning home BP. Masked hypertension was frequent (24.8%). Monotherapy was common (59.2%), while diuretics (10.1%) and beta-blockers (4.1%) were underutilized. A higher number of anti-hypertensive medications was associated with better BP control. Community-dwelling adults showed high hypertension prevalence, substantial untreated BP, and very low control rates under guideline targets. Home BP monitoring is essential for detecting uncontrolled morning and masked hypertension, and current treatment patterns appear insufficient to meet JSH recommendations.

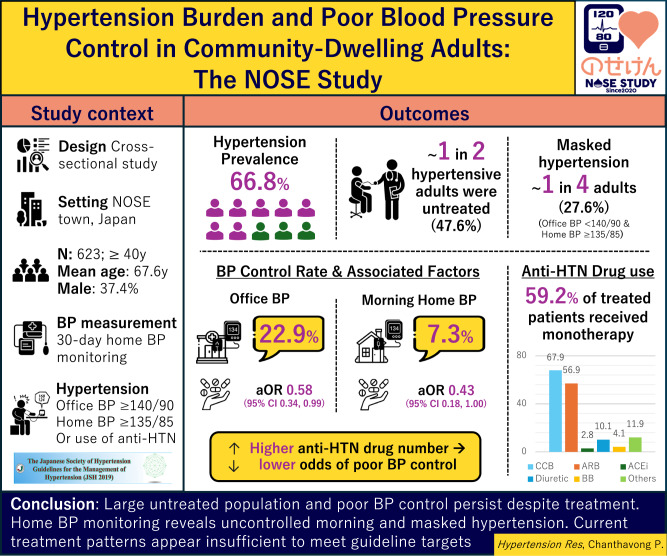

## Introduction

Hypertension is a leading modifiable risk factor for cardiovascular disease (CVD), stroke, and all-cause mortality worldwide [1, 2]. In Japan, it is a major public health challenge, accounting for approximately 100,000 deaths annually in the rapidly aging society [3]. Although national initiatives have improved hypertension awareness and treatment rates, which have exceeded 50% in recent years [4], blood pressure (BP) control remains suboptimal compared with that in high-income nations, indicating a persistent gap between treatment and effective management [5, 6].

The Japanese Society of Hypertension 2019 guidelines (JSH 2019) introduced more stringent BP targets for patients with hypertension, lowering the recommended office BP goal to <130/80 mmHg and <125/75 mmHg for home BP in most adults, except for those aged ≥75 years [4]. These lower thresholds reflect growing evidence that optimal BP control reduces cardiovascular morbidity and mortality [7]. A key feature of these guidelines is the out-of-office BP measurements integrations, particularly home BP monitoring. Traditional office BP measurement is limited by susceptibility to white-coat and masked hypertension, which may result in misclassification [8]. In contrast, home BP monitoring provides a more reliable assessment of an individual’s usual BP, enhances patient engagement, and is a superior predictor of cardiovascular outcomes [9–15]. Particularly, morning home BP is critical for identifying morning BP surges, a period of heightened cardiovascular risk [16, 17].

Despite these recommendations, the actual hypertension management status in the community, including the prevalence, treatment coverage, and control rates under stricter JSH 2019 targets, remains unclear, particularly in rural settings. Existing studies on BP status (hypertension prevalence and treatment) and control often rely on either office BP or short-term home BP monitoring (1–2 weeks) [18, 19], which may not fully reflect an individual’s long-term BP profile [20, 21]. This knowledge gap is particularly critical in communities such as Nose Town, which has higher CVD and stroke occurrence rates than the Japanese average [22–24], yet its local hypertension burden—a key underlying factor—remains unknown. This uncertainty hampers an accurate population burden assessment of uncontrolled or “hidden” hypertension under the revised criteria.

This study aimed to determine the actual BP status and control rate, evaluate the achievement of guideline-recommended targets, and identify the factors associated with hypertension, treatment and poor BP control in this community-dwelling Japanese population. Using baseline data from the NOSE Study, this study assessed BP status and control rates according to the JSH 2019 in both treated and untreated individuals to clarify the real-world hypertension burden.

Point of view

- Clinical relevance:
  A high prevalence of untreated and masked hypertension, as well as poor BP control—particularly morning home BP—in rural Asian communities highlights the urgent need for systematic home BP monitoring.
- Future direction:
  Longitudinal studies evaluating home BP-guided interventions are needed to improve early detection and long-term BP control in aging Asian populations.
- Consideration for the Asian population:
  Stronger BP–CVD associations, higher stroke risk, and a greater prevalence of morning BP surge in Asians emphasize the importance of home morning BP monitoring to enhance hypertension detection, improve BP control, and prevent cardiovascular events.

## Methods

### Study setting

Nose Town is a rural municipality located in northern Osaka Prefecture, Japan, with approximately 9000 residents as of 2022 [23, 25]. The population is markedly aged, with 43.4% of residents aged ≥65 years, substantially exceeding national (28.6%) and prefectural (27.0%) averages [22]. Socioeconomic indicators reflect a rural context, with modest income levels [26], lower educational attainment, and a mixed occupational structure characterized by higher engagement in agriculture and forestry compared with national averages [22, 27].

Healthcare resources are limited: the town has no inpatient hospital facilities, and primary care is provided through four outpatient clinics without hypertension specialty services [22, 28]. Municipal health statistics indicate a substantial burden of CVD, with cerebrovascular disease and heart failure accounting for a higher proportion of deaths than national and prefectural averages [22, 23]. These characteristics provide important context for interpreting hypertension prevalence, treatment patterns, and BP control in this community.

### Study population

Participants were recruited from the NOSE Study (UMIN-CRT ID: UMIN000039349), a community-based intervention study conducted in Nose Town, Osaka Prefecture, Japan. The NOSE Study’s primary aim is to assess whether long-term home BP monitoring could prevent cognitive decline, frailty, and CVD [29].

Recruitment was conducted between August 2020 and August 2021 through local newsletters, health checkups, community events, and COVID-19 vaccination sites. In total, 1151 community-dwelling adults aged ≥40 years participated in this study. Participants were allocated based on their residential district to either an early intervention group (monitored from 2020) or a late intervention group (monitored from 2022). Further details of the NOSE study protocol have been previously published [24, 29].

**Fig. 1:**
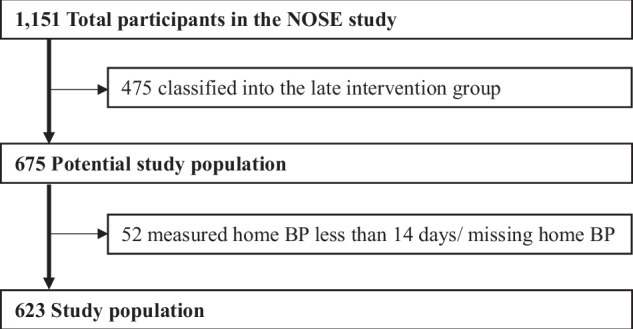
Schematic flow diagram of the selection of study participants. *BP* Blood Pressure

In the present cross-sectional analysis, only the early intervention group (*n* = 675) was included, and 52 individuals who had missing home BP data or did not meet the home BP measurement criteria (i.e., <14 days of measurements or initiation >30 days after enrollment) were excluded. The final sample consisted of 623 participants (Fig. 1). Among these participants, 579 (92.8%) recorded home BP measurements on 28–30 days. The mean number of measurement days was 29.4 ± 1.9 days (median 30; range 14–30).

The study was approved by the Ethics Committee of Osaka University Hospital (Approval No. 19433-4), and written informed consent was obtained from all participants.

### BP measurement

#### Office BP

Trained physicians or nurses measured office BP using an automated device (Omron HEM-7281T, Kyoto, Japan) at the community survey sites. Measurements were taken in the seated position after 2 min of rest in both arms, with two readings per arm. The average of the four measurements was used in the analysis.

#### Home BP

Participants were provided with the same automated device used at the survey site. At the time of enrollment, trained nurses provided education and instruction on self-measurement according to JSH 2019 [4]: twice in the morning (within 1-h of waking, after urination, before breakfast, and medication) and twice in the evening (before bedtime), using the same arm consistently. Each occasion included two consecutive readings, and the average was used for analysis. Only the first 30 days of the home BP data were used for this analysis to align with the baseline office measurements. The morning and evening home BP were analyzed separately.

### Definitions of hypertension, BP status, and BP control

“BP status” is used as a descriptive term referring to the presence of hypertension and treatment status (i.e., non-hypertension, untreated and treated hypertension), and was conceptually distinct from BP control, which refers to achievement of guideline-defined BP targets among treated participants.

- **Non-hypertension:** Defined as participants not using any anti-hypertensive medications who had both office BP < 140/90 mmHg and morning home BP < 135/85 mmHg.
- **Hypertension**: Defined as participants who met either of the following criteria: Office BP ≥ 140/90 mmHg, or Morning home BP ≥ 135/85 mmHg, regardless of anti-hypertensive medication use. Hypertension was further subclassified as:
  - **Untreated Hypertension:** Defined as participants not using any anti-hypertensive medications who had office BP ≥ 140/90 mmHg or morning home BP ≥ 135/85 mmHg.
  - **Treated hypertension:** Defined as participants using one or more anti-hypertensive medications, irrespective of BP levels.
- **White-coat hypertension:** Defined as office BP ≥ 140/90 mmHg and morning home BP < 135/85 mmHg.
- **Masked hypertension:** Defined as office BP < 140/90 mmHg and morning home BP ≥ 135/85 mmHg.

### BP control

BP control was defined using individualized target values based on the Japanese Society of Hypertension 2019 guidelines (JSH 2019) [4]. Target BP thresholds were determined according to age and the presence of major clinical comorbidities that were reliably available in the study dataset.

For office BP, a target of <130/80 mmHg was applied to participants aged <75 years and to those with high-risk comorbidities (diabetes mellitus, chronic kidney disease (CKD), established CVD, or use of antithrombotic drugs). A target of <140/90 mmHg was applied to participants aged ≥75 years without these high-risk conditions.

For home BP, target values were defined as 5 mmHg lower than the corresponding office BP targets, in accordance with JSH 2019 guidance.

A participant was classified as having controlled BP if their measured BP was below the applicable individualized target for the BP measurement modality analyzed (office BP or morning home BP). Poor BP control was defined as BP equal to or exceeding the corresponding target.

All analyses involving home BP control were based on morning home BP measurements only.

To assess the robustness, BP control rates based on the updated JSH 2025 guidelines [30] were additionally calculated as a supplementary analysis.

### Covariates

Covariates were selected a priori based on clinical relevance and prior hypertension epidemiology. Sociodemographic and lifestyle variables included age (years, continuous), sex (male/female), educational level (< 12 vs. ≥12 years), living arrangement (living alone vs not), smoking status (non-smoker vs current or former smoker), and alcohol consumption (non-drinker vs drinker).

Body mass index (BMI) was categorized as underweight (< 18.5 kg/m²), normal weight (18.5–24.9 kg/m²), and overweight ( ≥ 25.0 kg/m²), while BMI was modeled as a continuous variable in multivariable regression analyses.

Clinical covariates included diabetes mellitus, CKD, dyslipidemia, and history of CVD. History of CVD was defined as a composite of cerebrovascular disease or coronary heart disease. CKD was defined based on estimated glomerular filtration rate values obtained from participants’ routine health checkup records.

Medication-related variables included use of antithrombotic drug (yes/no), and the number of anti-hypertensive drug classes prescribed (continuous).

Not all covariates were included in every multivariable model; covariate inclusion varied by outcome and is detailed in the Statistical Analysis section.

Detailed diagnostic criteria for comorbid conditions and classifications of anti-hypertensive medications are provided in the Supplementary Materials.

### Statistical analysis

Continuous variables are presented as mean ± standard deviation (SD), and categorical variables as frequencies or percentages. Between-group comparisons across the three BP groups were performed using one-way analysis of variance (ANOVA) for normally distributed continuous variables and the Kruskal–Wallis test for non-normally distributed continuous variables. Categorical variables were compared using the chi-squared test or Fisher’s exact test, as appropriate.

Covariate selection was prespecified based on prior hypertension epidemiology and JSH 2019 guidelines to adjust for key confounders while avoiding over-adjustment. Separate multivariable models were constructed for each outcome using outcome-specific covariate sets appropriate to the underlying study population and research question.

For hypertension prevalence, Poisson regression with robust variance estimation was used, adjusting for age, sex, BMI, smoking status, alcohol consumption, educational level, living arrangement, diabetes mellitus, and CKD.

For receipt of anti-hypertensive treatment among participants with hypertension, Poisson regression with robust variance estimation was used, adjusting for the above covariates with the addition of dyslipidemia, history of CVD and antithrombotic use.

For poor BP control among treated participants, multivariable logistic regression was used, additionally adjusting for the number of prescribed anti-hypertensive drug classes as a marker of treatment intensity.

Multicollinearity among covariates was assessed using variance inflation factors (VIFs), and no evidence of problematic multicollinearity was observed in the final models. Statistical significance was defined as a two-tailed *p*-value < 0.05. All analyses were performed using IBM SPSS Statistics version 28.0 (IBM Japan, Tokyo, Japan).

## Results

### Baseline characteristics

The baseline characteristics of the 623 participants, stratified according to hypertension status and treatment, are presented in Table 1. The overall prevalence of hypertension was 66.8% (416/623), and among those with hypertension, 52.4% (218/416) were receiving anti-hypertensive treatment.

**Table 1:** Baseline characteristics of the total population (*N* = 623)

		Non-Hypertension (*N* = 207)	Untreated Hypertension (*N* = 198)	Treated Hypertension (*N* = 218)	*P*-value
Mean age, years		63.6 ± 10.1	67.9 ± 9.2	71.1 ± 8.9	**<0.001**
Age group, years	**40- < 65, %**	50.7	29.8	19.7	**<0.001**
	**65 - < 75, %**	37.7	47.5	46.3	
	**≥ 75, %**	11.6	22.7	33.9	
Male, %		24.0	33.9	42.1	**<0.001**
Mean BMI, kg/m^2^		22.4 ± 3.0	23.7 ± 3.5	24.4 ± 3.2	**<0.001**
BMI categories, kg/m^2^	**Underweight, %**	6.8	3.0	2.3	**<0.001**
	**Normal, %**	75.2	64.6	57.1	
	**Overweight, %**	18.0	32.3	40.6	
Office	**SBP, mmHg**	118.6 ± 11.6	144.8 ± 16.6	139.7 ± 17.1	**<0.001**
	**DBP, mmHg**	75.0 ± 7.1	88.4 ± 10.9	82.6 ± 10.2	**<0.001**
	**PR, bpm**	70.9 ± 9.7	74.2 ± 10.9	74.0 ± 11.0	**0.002**
Morning Home	**SBP, mmHg**	117.5 ± 9.6	138.9 ± 13.4	135.3 ± 12.5	**<0.001**
	**DBP, mmHg**	74.4 ± 6.2	86.2 ± 8.6	81.8 ± 9.2	**<0.001**
	**PR, bpm**	65.4 ± 7.8	66.5 ± 9.0	65.6 ± 8.3	0.340
Evening Home	**SBP, mmHg**	112.3 ± 9.6	130.1 ± 11.9	127.2 ± 12.3	**<0.001**
	**DBP, mmHg**	69.8 ± 6.6	79.5 ± 8.6	75.2 ± 8.7	**<0.001**
	**PR, bpm**	68.5 ± 8.6	70.7 ± 8.9	69.9 ± 9.2	**0.036**
Living alone, %		10.1	9.1	17.0	**0.027**
Alcohol consumption, %		45.4	56.1	46.8	**0.066**
Educational level≤12years, %		52.2	60.6	67.4	**0.006**
Current or ex-smoker, %		25.6	40.4	39.4	**0.002**
Diabetes, %		5.3	13.1	22.9	**<0.001**
Dyslipidemia, %		50.2	52.5	71.6	**<0.001**
History of CVD, %		3.9	3.5	16.5	**<0.001**
Chronic Kidney Disease, %		13.5	15.7	29.4	**<0.001**
Antithrombotic drug, %		1.9	1.0	6.0	**<0.007**

Data are presented as mean ± standard deviation or percentage. Group differences were assessed using one-way analysis of variance for continuous variables and chi-squared test or Fisher’s exact test, as appropriate, for categorical variables. *P*-values  <  0.05 were considered statistically significant. BMI categories were defined as underweight ( < 18.5 kg/m²), normal weight (18.5–24.9 kg/m²), and overweight ( ≥ 25.0 kg/m²). Educational level ≤ 12 years indicates completion of less than high school education. History of cardiovascular disease (CVD) includes self-reported cerebrovascular disease or coronary heart disease. Atrial fibrillation was assessed by self-report; however, due to the small number of reported cases and potential under-ascertainment, antithrombotic drug use based on prescription records is presented. *BP* Blood Pressure, *BMI* Body mass index, *SBP* Systolic Blood Pressure, *DBP* Diastolic Blood Pressure, *PR* Pulse Rate, *BMI* Body Mass Index, *CVD* Cardiovascular disease

Significant differences were observed among the three groups with respect to age, sex distribution, BMI, BP levels, and comorbidity prevalence (all *p* < 0.001). Participants in the treated hypertension group tended to be older (mean age 71.1 ± 8.9 years) and had higher BMI values (24.4 ± 3.2 kg/m²).

BP levels varied across groups. The untreated hypertension group had the highest mean BP across all measurement modalities (office BP: 144.8/88.4 mmHg), followed by the treated hypertension group (139.7/82.6 mmHg) and the non-hypertension group (118.6/75.0 mmHg). Similar patterns were observed for morning and evening home BP.

Lifestyle factors and comorbidities, including smoking history, diabetes, CKD, and history of CVD, also differed significantly across groups.

### Factors associated with having hypertension among the overall population

The factors associated with having hypertension in the overall population are shown in Supplementary Table 1. In the multivariable Poisson regression analysis, older age and higher BMI were independently associated with a higher prevalence of hypertension. Each one-year increase in age was associated with a 2% higher prevalence of hypertension (prevalence ratio [PR] = 1.02, 95% confidence interval [CI] 1.01–1.04), and each 1 kg/m² increase in BMI was associated with a 5% higher prevalence (PR = 1.05, 95% CI 1.02–1.08). No other covariates were significantly associated with hypertension prevalence after adjustment.

### Factors associated with receiving treatment among population with hypertension

The factors associated with anti-hypertensive treatment among the 416 participants with hypertension are shown in Supplementary Table 2. In the multivariable Poisson regression analysis, dyslipidemia (PR = 1.42, 95% CI 1.05–1.92) and a history of CVD (PR = 1.49, 95% CI 1.01–2.18) were significantly associated with receiving treatment. No other covariates showed statistically significant associations.

### BP control and hypertension phenotypes

White-coat and masked hypertension prevalences are shown in Supplementary Fig. 1. Overall, white-coat hypertension was identified in 18.3% of the population, whereas masked hypertension was present in 27.2%. The prevalence of both conditions was higher in the untreated hypertensive participants than in treated group.

**Fig. 2:**
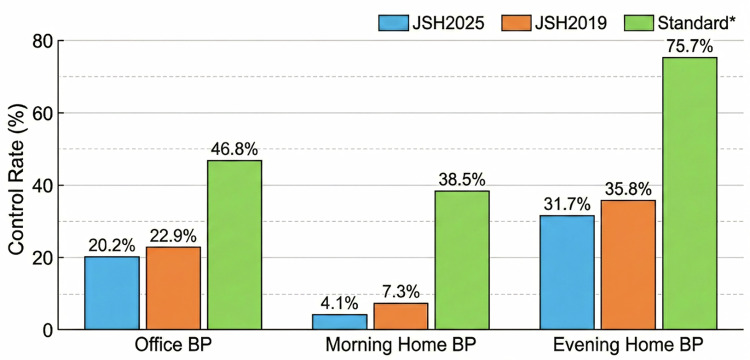
Blood Pressure Control Rate among treated hypertensive participants according to different blood pressure targets (*N* = 218) Data are presented as percentages of participants achieving blood pressure (BP) control under each criterion. BP control rates were assessed using office BP, morning home BP, and evening home BP measurements. Control rate was defined according to the Japanese Society of Hypertension (JSH) 2019 guidelines, the JSH 2025 guidelines, and standard BP thresholds (office BP < 140/90 mmHg; home BP < 135/85 mmHg). *BP* Blood Pressure, *JSH* Japanese Society of Hypertension

Among the 218 participants with treated hypertension, the BP control rates varied according to the diagnostic criteria (Fig. 2). Office BP control rate was 46.8% (< 140/90 mmHg), but 22.9% under the JSH 2019 targets. Morning home BP control rates were 38.5% (< 135/85 mmHg) and 7.3% (JSH 2019 targets). Evening home BP control rates were 75.7% and 35.8%, respectively.

### Anti-hypertensive medication usage

Anti-hypertensive medication patterns use among the 218 participants with treated hypertension are shown in Table 2 and Supplementary Fig. 2. The mean number of prescribed anti-hypertensive agents was 1.4 ± 0.7, and fixed-dose combinations were used in 13.3% of patients. Morning dosing was prescribed in 81.2%.

**Table 2:** Anti-hypertensive medication use among participants with treated hypertension

Variables	Participants with treated hypertension
	*N* = 218
Mean number of anti-hypertensive agents	1.4 ± 0.7
Fix-dose combination Usage, %	13.3
Medication Timing (morning), %	81.2
Medication Therapy Regime	
Monotherapy, %	59.2
2-drug combination, %	30.3
≥ 3-drug combination, %	10.6
Overall Drug Class Usage	
Calcium Channel Blockers, %	67.9
Angiotensin II Receptor Blockers, %	56.9
Angiotensin-Converting Enzyme, %	2.8
Diuretic, %	10.1
Beta-blocker, %	4.1
Alpha-beta-blocker, %	4.6
Mineralocorticoid Receptor Antagonists, %	1.8
Alpha-blocker, %	3.2
Others, %	2.3

Data are presented as mean ± standard deviation or number (percentage). Fixed-dose combination use refers to single pills containing two or more anti-hypertensive drug classes. The frequency of each anti-hypertensive drug class is expressed as the proportion of patients receiving anti-hypertensive treatment

Regarding treatment regimen, monotherapy was used by 59.2% of patients, while 40.8% received combination therapy, including 30.3% on two-drug combination and 10.6% on ≥ 3 agents. The most frequently prescribed drug classes were calcium channel blockers (67.9%), followed by angiotensin II receptor blockers (56.9%), diuretics (10.1%), and beta-blockers (4.1%).

### Factors associated with poor BP control

Among treated participants, factors associated with poor BP control are shown in Table 3. Poor office BP control was significantly associated with the presence of CKD (adjusted odds ratio [aOR] 3.49, 95% CI 1.38–8.83), whereas living alone (aOR 0.35, 95% CI 0.14–0.84) and using a higher number of anti-hypertensive drugs (aOR 0.58, 95% CI 0.34–0.99) were associated with lower odds of poor control.

**Table 3:** Factors associated with Poor BP control in treated hypertensive participants (*N* = 218, based on JSH 2019)

Variables		Office BP	Morning Home BP	Evening Home BP
		Odds ratios (95% CI)	Odds ratios (95% CI)	Odds ratios(95% CI)
Common factors				
Gender (ref: male)	Female	1.05 (0.37, 3.04)	0.32 (0.05, 2.14)	1.30 (0.54, 3.15)
Age		0.98 (0.94, 1.02)	0.95 (0.88, 1.02)	0.96 (0.92, 0.99)*
Body mass index		1.08 (0.96, 1.21)	0.97 (0.80, 1.17)	1.02 (0.92, 1.13)
Alcohol Consumer (ref: no)	Yes	1.59 (0.75, 3.35)	0.35 (0.10, 1.19)	0.59 (0.31, 1.14)
Educational Group (ref: ≤ 12y)		0.59 (0.28, 1.29)	0.45 (0.12, 1.62)	0.78 (0.39, 1.54)
Living alone (ref: no)	Yes	0.35 (0.14, 0.84)*	0.27 (0.07, 1.03)	0.90 (0.38, 2.16)
Current or ex-smoker (ref: non-smoker)	Yes	1.32 (0.47, 3.69)	1.79 (0.27, 11.98)	2.41 (1.00, 5.79)*
Disease History				
Diabetes (ref: no)	Yes	0.94 (0.39, 2.23)	0.62 (0.15, 2.51)	1.79 (0.81, 3.88)
Dyslipidemia (ref: no)	Yes	0.94 (0.43, 2.04)	0.32 (0.06, 1.58)	0.81 (0.41, 1.61)
History of cardiovascular disease (ref: no)	Yes	1.45 (0.56, 3.80)	4.46 (0.51, 38.95)	2.69 (1.03, 7.05)*
Chronic kidney disease (ref: no)	Yes	3.49 (1.38, 8.83)*	2.01 (0.45, 9.03)	1.64 (0.78, 3.44)
Antithrombotic user (ref: no)	Yes	1.04 (0.22, 4.78)	2.08 (0.09, 43.67)	7.48 (0.78, 71.84)
Number of anti-hypertensive drug used		0.58 (0.34, 0.99)*	0.43 (0.18, 1.00)*	1.09 (0.65, 1.83)

Odds ratios (ORs) and 95% confidence intervals (CIs) were estimated using multivariable logistic regression analyses. Separate models were constructed for office BP, morning home BP, and evening home BP. Poor BP control was defined as BP equal to or exceeding the individualized target value specified by the JSH 2019 guidelines for the corresponding measurement modality. Age (years), body mass index (kg/m²), and number of anti-hypertensive drug classes were included as continuous variables. Alcohol consumption was categorized as non-drinker or drinker. Educational level was categorized as ≤12 or >12 years of education. Smoking status was categorized as non-smoker or current/former smoker. Living arrangement was categorized as living alone or not. Diabetes, dyslipidemia, chronic kidney disease, and history of cerebrovascular disease were defined as described in the Methods section. All variables shown were entered simultaneously into each multivariable model

*P < 0.05. *BP* blood pressure, *CI* confidence interval, *JSH* Japanese Society of Hypertension

For morning home BP, a higher number of anti-hypertensive drugs were associated with lower odds of poor control (aOR 0.43, 95% CI 0.18–1.00). For evening home BP, current or past smoking (aOR 2.41, 95% CI 1.00–5.79) and a history of CVD (aOR 2.69, 95% CI 1.03–7.05) were associated with higher odds of poor evening BP control.

## Discussion

In this community-based study of Japanese adults, a multi-layered problem in hypertension management was identified. Hypertension prevalence was markedly high, exceeding prior national surveys and community-based studies [5, 31]. Despite this high burden, only about half of individuals who met the diagnostic criteria for hypertension were receiving anti-hypertensive treatment (Table 1). Although treatment coverage among individuals with hypertension was broadly comparable to national averages [4, 5, 31], a considerable proportion of affected individuals remained untreated, underscoring a persistent public health challenge in this rural community.

To clarify the factors underlying the high burden of hypertension in this community, we examined population-level patterns of hypertension prevalence (Supplementary Table 1). Hypertension in Nose Town largely reflected broader demographic and lifestyle transitions, with aging and excess body weight emerging as dominant contributors, consistent with prior evidence [32–34]. These results suggest that hypertension in this rural setting is driven less by clinically recognized disease and more by gradual, cumulative exposures that operate across the life course. This highlights the importance of early and population-wide prevention strategies, particularly weight control and routine BP assessment.

A particularly important finding of this study is the high prevalence of untreated hypertension in this rural community. Nearly half of individuals meeting diagnostic criteria for hypertension were not receiving anti-hypertensive treatment, a proportion consistent with national surveys [4] and prior population-based studies in Japan and internationally [35, 36]. These findings indicate that untreated hypertension remains a pervasive public health challenge, including in rural communities.

Several contextual factors may contribute to the high untreated rate observed in Nose Town. National data indicate that participation in community-based health checkup programs remains suboptimal, and follow-up health guidance is delivered to only a minority of individuals identified as requiring intervention [4]. In this rural setting—characterized by few clinics and the absence of hospital-based care—opportunities for BP screening, diagnosis, and treatment initiation may be further constrained [22, 28]. Consequently, a reliance on office BP measurements may contribute to under-recognition of hypertension, a limitation commonly encountered in rural regions of Japan [37].

Comparison between treated and untreated individuals with hypertension revealed a clinically meaningful and potentially overlooked subgroup (Supplementary Table 3). Those with untreated hypertension tended to have fewer diagnosed comorbidities yet exhibited higher BP levels across both office and home measurements.

This apparent paradox likely reflects differences in disease recognition and health-seeking behavior rather than lower underlying cardiovascular risk. In our study, a substantial proportion of untreated individuals were aged the 40–65-years, corresponding to the working-age population, who may have fewer healthcare encounters due to occupational demands [36, 38]. The absence of overt comorbidities, combined with the largely asymptomatic nature of hypertension [39], may further reduce perceived urgency for medical evaluation.

These barriers may be further amplified in rural settings [22, 27], where employment is often concentrated in primary and secondary industries and access to structured workplace health examinations is limited [4, 40]. As a result, younger individuals without manifest disease may be more likely to delay formal diagnosis and rely on non-clinical health information sources, such as online resources [41], contributing to delayed treatment initiation.

Untreated hypertension in this group may reflect early-stage or masked hypertension, conditions in which office BP measurements underestimate true BP burden. While salt sensitivity [42] and high dietary salt intake [43] are well-recognized contributors to hypertension in Japan, behavioral factors, psychosocial stress, and work-related demands may play a relatively larger role in BP elevation among younger populations.

Consistent with these observations, factors associated with receiving anti-hypertensive treatment appeared to reflect healthcare contact driven by established cardiovascular morbidity rather than elevated BP alone (Supplementary Table 2). Individuals with dyslipidemia or a history of CVD were more likely to receive treatment, whereas age itself was no longer independently associated after adjustment. This pattern suggests that hypertension may often be identified incidentally during clinical encounters for comorbid conditions, rather than through systematic BP screening. This reliance on opportunistic detection further highlights the limitations of office-based assessment. It also provides important context for the high prevalence of masked hypertension observed in this community.

The high burden of discordant BP phenotypes observed in this study highlights the limitations of relying solely on office BP measurements in community settings (Supplementary Fig. 2). In particular, masked hypertension was frequently identified (27.2%), especially among individuals who were not receiving anti-hypertensive treatment, suggesting that a substantial proportion of hypertension remains clinically unrecognized when assessment is limited to clinic-based measurements. This finding provides a plausible explanation for the persistent gap between hypertension prevalence and treatment coverage observed in this population.

The prevalence of masked hypertension in the NOSE Study was higher than that reported in previous Japanese and international population-based studies, which have generally reported prevalences of approximately 10–20% [44–48].

Several factors may account for these differences. First, the NOSE Study population was markedly older, with nearly 60% of participants aged ≥65 years, whereas many prior studies included younger populations or a smaller proportion of older adults [46, 47]. Given that masked hypertension is more prevalent with advancing age [49], the demographic structure likely contributed to the higher prevalence observed. Second, differences in BP measurement protocols may also have influenced detection; repeated home BP measurements over up to 30 days may have improved detection compared with prior studies that relied on shorter measurement periods, potentially reducing random variability and underestimation [8]. In addition, the rural setting of Nose Town [22, 28] may contribute to delayed diagnosis and a higher burden of untreated hypertension compared with urban populations.

In contrast, white-coat hypertension was observed predominantly among treated individuals (16.5%), raising concern about potential overtreatment in this subgroup. These findings underscore the critical role of home BP monitoring in accurately characterizing BP phenotypes and guiding appropriate treatment decisions, particularly in aging and rural populations where discordant BP patterns are common.

Beyond hypertension diagnosis and treatment initiation, the analysis of the 218 participants receiving anti-hypertensive medication revealed that BP control remained suboptimal (Fig. 2). Application of contemporary guideline targets revealed a substantial decline in control rates compared with conventional thresholds, highlighting the gap between real-world practice and increasingly stringent treatment goals.

Using traditional criteria, office BP control in our cohort was broadly comparable to earlier Japanese community-based studies (e.g., J-HOME and Ohasama) [18, 50, 51]. However, application of the JSH 2019 targets [4] resulted in markedly lower control rates, consistent with recent reports adopting stricter BP definitions [19, 52]. A more pronounced discrepancy was observed for morning home BP, where control rates decreased sharply under lower target levels (Fig. 2). Similar findings have been reported in previous studies [51, 52]. This reduction likely reflects the combined impact of lower numerical thresholds, the complex, risk-stratified approach of the guidelines, and the physiological challenge of the morning BP surge [4, 34, 53].

Although this study was designed under the JSH 2019 framework, re-evaluation using the recently published JSH 2025 [30] criteria yielded similarly low control rates. These findings indicate that the observed reduction in BP control reflects not only the progressive tightening of guideline targets but also persistent challenges in achieving adequate BP control in routine clinical practice.

To better understand the mechanisms underlying these low control rates, we examined factors associated with poor BP control across different measurement settings (Table 3). Determinants differed across office, morning home, and evening home BP, indicating that BP control is shaped by measurement context, circadian physiology, and treatment dynamics rather than measurement inconsistency.

Morning home BP, assessed under highly standardized conditions and prior to medication intake [4], reflects BP at the pharmacodynamic trough and captures the morning BP surge [34, 53]. Accordingly, the association between a greater number of anti-hypertensive drug classes (Table 3) and better morning BP control likely indicates the need for sufficient treatment intensity and sustained 24-h drug efficacy. This provides a plausible explanation for the particularly low morning BP control rates observed in this study.

In contrast, evening home BP was measured before bedtime without strict control for factors such as meal timing, bathing, alcohol consumption, or physical activity. In the Japanese context, evening behaviors such as bathing [54] and alcohol intake [55] are common and have been shown to transiently lower BP, potentially increasing variability and attenuating associations with clinical determinants. These protocol differences likely contribute to the weaker and more heterogeneous associations observed for evening BP compared with morning BP.

Office BP, by contrast, may be more susceptible to situational influences such as white-coat effects, particularly among patients with CKD, in whom volume-dependent and vascular mechanisms may amplify BP variability [56]. Consistent with this, CKD (aOR 3.49) emerged as a strong determinant of poor office BP control.

An unexpected finding was that living alone was associated with better office BP (aOR 0.35). This contrasts with previous reports linking solitary living to poorer BP control [57, 58]. In the present cohort, this association may reflect population-specific characteristics, including the older age distribution among participants living alone, for whom less stringent BP targets are recommended under JSH guidelines, as well as potential selection of relatively health-conscious individuals among community-dwelling volunteers. However, residual confounding cannot be excluded, and this finding should be interpreted cautiously.

Overall, these findings indicate that factors associated with BP control differ meaningfully according to measurement timing and context. Morning and evening home BP should therefore be viewed as distinct BP phenotypes, influenced by different physiological, behavioral, and treatment-related factors. These results underscore the importance of multi-modal BP assessment and phenotype-specific interpretation when evaluating treatment adequacy and guiding individualized anti-hypertensive management.

The medication prescription patterns in the treated population offer further insights into suboptimal control rates (Table 2; Supplementary Fig. 2). While calcium channel blockers and angiotensin II receptor blockers’ dominance aligns with the guideline recommendations [4], several findings point to significant clinical inertia. Most patients (59.2%) were on monotherapy, and fixed-dose combinations were low (13.3%). Given that combination therapy is approximately five times more effective than doubling the monotherapy dose [59], the reluctance to intensify therapy represents a major potential contributor to treatment failure. This pattern likely reflects clinical inertia in the non-specialist, rural care setting of Nose town [22, 28], where physicians may rely more on office BP readings, which may appear acceptable, and being less familiar with the benefits of adherence-promoting fixed-dose combinations [12, 60–63]. This interpretation was supported by the regression analysis, which showed that a higher number of medications was significantly associated with better BP control (Table 3).

Furthermore, the underutilization of key drug classes and the questionable prescription hierarchy were notable. Diuretics, a recommended first-line agent and the cornerstone of intensive treatment regimens, were used in only 10.1% of the patients [64]. This may be due to their infrequent use as a single agent or a potential physician concern for side effects in the Japanese population, which is known to be highly salt-sensitive [65]. Similarly, beta-blockers were prescribed to only 4.1% of patients (despite a 16.5% prevalence of CVD), whereas later-line agents such as alpha-beta blockers (4.6%) and alpha-blockers (3.2%) were used at similar or higher rates, suggesting that prescription decisions may not strictly follow the stepwise therapeutic algorithm recommended by the guidelines [4].

Finally, medication timing suggests that current prescription patterns may not adequately address morning BP surges [34, 53]. In our cohort, 81.2% of patients received morning-only dosing. Although the long-term cardiovascular benefits of chronotherapy remain controversial [66], this traditional practice provides a plausible mechanism for the poor morning control observed, as the therapeutic effect of many agents may wane overnight [67]. This prescription pattern likely reflects clinical caution regarding nocturnal hypotension and risk of falls in older populations [68]. Regardless, the current treatment strategy, combined with a treatment rate of only 50% despite its prevalence, is clearly insufficient for controlling BP, especially morning home BP.

This study has several notable strengths, including a real-world evaluation based on the JSH 2019 guidelines, comprehensive multi-modal BP assessment using office and 30-day home measurements (morning and evening), clearly defined BP targets according to comorbidity status, and a focus on a community-dwelling rural population that is often underrepresented in hypertension research.

Nevertheless, several limitations should be acknowledged. First, the cross-sectional design precludes any causal inference. Second, home BP values were manually recorded by participants, and lifestyle variables were self-reported, introducing the possibility of measurement error and reporting bias. In addition, participation was voluntary, which may have resulted in a healthy participant bias. Third, morning home BP was measured before medication intake, which provides an unmedicated estimate but may partially reflect the pharmacodynamic trough of prior anti-hypertensive therapy [67].

Fourth, the analysis lacked detailed information on several potentially important determinants, including medication adherence, dietary salt intake, and other lifestyle factors. Fifth, antithrombotic drug use likely reflected heterogeneous underlying conditions, and limited data on atrial fibrillation and antithrombotic subtypes may have resulted in residual confounding. Finally, physician specialty information at the individual level was unavailable, precluding analyses stratified by provider type.

Although data collection occurred during the JSH 2019 era, complementary analyses using JSH 2025 criteria demonstrated that the observed gaps in BP status and control persist under contemporary guideline thresholds, supporting the ongoing clinical relevance of our findings.

As the NOSE study is an ongoing prospective cohort, future longitudinal analyses will enable evaluation of BP trajectories over time, assessment of causal relationships, and investigation of the real-world impact of home BP monitoring–based interventions on hypertension management.

### Perspective of Asia

BP management in Asia presents distinct challenges. The association between elevated BP and CVD risk is stronger in Asian populations than in Western populations [69], particularly for stroke, which remains a leading cause of long-term disability in Asia [70]. In addition, morning BP surge and masked hypertension are more frequently observed in Asian individuals [71], further increasing the risk of cerebrovascular events [34, 53]. These characteristics underscore the importance of home BP monitoring—especially morning measurements—in Asian populations.

By focusing on a rural, community-dwelling population in Nose Town, this study addresses a setting underrepresented in hypertension research yet increasingly common across Asia, where aging demographics, limited healthcare access, and suboptimal implementation of guideline-based care coexist [12, 37, 62, 63]. The high prevalence of untreated hypertension, masked hypertension, and poor morning BP control observed in this study reveals substantial gaps in detection and management. Promoting systematic home BP monitoring is therefore essential to improve hypertension detection, optimize treatment strategies, enhance BP control, and reduce stroke burden in rapidly aging Asian societies.

## Conclusion

In conclusion, this study revealed a substantial unmet need for hypertension management, characterized by a high prevalence of hypertension (66.8%) and a treatment rate of approximately 50%. Among those receiving treatment, BP control was poor under the JSH 2019, with home BP monitoring identifying a substantial hidden and uncontrolled hypertension burden. These findings indicate significant clinical inertia in treatment intensification as a key driver of these poor outcomes. To improve BP control rates, the results underscore two essential actions: first, the broader home BP monitoring implementation is necessary to accurately identify and manage uncontrolled hypertension, and second, disseminating the knowledge and recommendations of the latest hypertension guidelines to all people and healthcare professionals is critical to bridge the gap between evidence and real-world practice.

## Supplementary information

Supplementary Methods
