# Supplementary material for: Blood pressure status, JSH 2019-based control rate, and associated factors among community-dwelling adults: The NOSE study
Source: Hypertens Res. 2026 Apr 6;49(6):1853–65. doi: 10.1038/s41440-026-02622-8 (PMC13236594; doi:10.1038/s41440-026-02622-8)
Supplement: Supplementary file 1 — Supplementary Methods [file 41440_2026_2622_MOESM1_ESM.docx]

# **Supplementary Methods:**

- **Definitions of Comorbidities and Medications:**

Medical history and anti-hypertensive medications were obtained from participant health diaries and questionnaires:

- Diabetes mellitus: Antidiabetic medication use or fasting glucose ≥126 mg/dL or random glucose ≥200 mg/dL.
- Chronic kidney disease: Estimated glomerular filtration rate (eGFR) <60 mL/min/1.73 m². eGFR values were obtained directly from participants’ routine health checkup records and were not recalculated by the investigators. These values were originally calculated at the health checkup facilities according to the Japanese Society of Nephrology (JSN) equation based on serum creatinine.
- Dyslipidemia: Use of lipid-lowering medication or triglycerides ≥150 mg/dL or LDL-C ≥140 mg/dL.
- Cerebrovascular disease: History of cerebral infarction, cerebral hemorrhage, or subarachnoid hemorrhage.
- Coronary heart disease: History of angina pectoris or myocardial infarction.
- History of cardiovascular disease: Defined as a composite variable indicating the presence of either cerebrovascular disease or coronary heart disease. This combined variable was used in multivariable regression analyses to improve model stability.
- Antithrombotic drug use: Use of antiplatelet or anticoagulant agents identified from medication records.

Anti-hypertensive medication: Included calcium channel blockers, angiotensin II receptor blockers, angiotensin-converting enzyme, diuretics, beta-blockers, alpha-beta-blockers, alpha-blockers, mineralocorticoid receptor antagonists, and fixed-dose combinations.

Supplementary Table 1 Factors associated with hypertension in the overall population (N=623)

| Variables |  | Having hypertension |
| --- | --- | --- |
|  |  | **Prevalence Ratio (95% CI)** |
| Gender (ref: male) | Female | 0.96 (0.74, 1.24) |
| Age |  | **1.02 (1.01, 1.04)**** |
| Body mass index |  | **1.05 (1.02, 1.08)**** |
| Alcohol Consumer (ref: no) | Yes | 1.10 (0.89, 1.36) |
| Educational Group (ref: ≤12y) | Yes | 0.99 (0.80, 1.23) |
| Living alone (ref: no) | Yes | 1.07 (0.79, 1.43) |
| Current or ex-smoker (ref: non-smoker) | Yes | 1.11 (0.87, 1.43) |
| Diabetes (ref: no) | Yes | 1.13 (0.86, 1.49) |
| Chronic kidney disease (ref: no) | Yes | 0.99 (0.77, 1.26) |

Prevalence ratios (PRs) and 95% confidence intervals (CIs) were estimated using multivariable Poisson regression with robust variance.
Hypertension was defined according to the study criteria.
Age (years) and body mass index (kg/m²) were included as continuous variables. Alcohol consumption was categorized as non-drinker or drinker. Educational level was categorized as ≤12 or >12 years of education. Smoking status was categorized as non-smoker or current/former smoker. Living arrangement was categorized as living alone or not.
Diabetes and chronic kidney disease were defined as described in the Methods section.
All variables shown were entered simultaneously into the multivariable model. *P < 0.05; **P < 0.01.
Abbreviations: *CI*, confidence interval.

Supplementary Table 2 Factors associated with receiving treatment in the population with hypertension (N=416)

| Variables |  | Receiving treatment for Hypertension |
| --- | --- | --- |
|  |  | **Prevalence Ratio (95% CI)** |
| Gender (ref: male) | Female | 0.82 (0.56, 1.18) |
| Age |  | 1.03 (0.99, 1.03) |
| Body mass index |  | 1.03 (0.98, 1.07) |
| Alcohol Consumer (ref: no) | Yes | 0.92 (0.69, 1.23) |
| Educational Group (ref: ≤12y) | Yes | 0.91 (0.67, 1.23) |
| Living alone (ref: no) | Yes | 1.14 (0.77, 1.68) |
| Current or ex-smoker (ref: non-smoker) | Yes | 0.84 (0.59, 1.20) |
| Diabetes (ref: no) | Yes | 1.20 (0.85, 1.69) |
| Dyslipidemia (ref: no) | Yes | **1.42 (1.05, 1.92)*** |
| History of cardiovascular disease (ref: no) | Yes | **1.49 (1.01, 2.18)*** |
| Chronic kidney disease (ref: no) | Yes | 1.19 (0.87, 1.63) |
| Antithrombotic user (ref: no) | Yes | 1.30 (0.72, 2.37) |

Prevalence ratios (PRs) and 95% confidence intervals (CIs) were estimated using multivariable Poisson regression with robust variance.
The outcome variable was current receipt of anti-hypertensive treatment.
Age (years) and body mass index (kg/m²) were included as continuous variables. Alcohol consumption was categorized as non-drinker or drinker. Educational level was categorized as ≤12 or >12 years of education. Smoking status was categorized as non-smoker or current/former smoker. Living arrangement was categorized as living alone or not.
Diabetes, dyslipidemia, chronic kidney disease, and history of cardiovascular disease were defined as described in the Methods section. History of cardiovascular disease was defined as a composite of cerebrovascular disease or coronary heart disease.
All variables shown were entered simultaneously into the multivariable model. *P < 0.05.
Abbreviations: *CI*, confidence interval.

Supplementary Table 3 Baseline characteristics of untreated and treated participants with hypertension (N=416)

| Variables | | Untreated Hypertension (N=198) | Treated Hypertension (N=218) | P-value |
| --- | --- | --- | --- | --- |
| Mean age, years | | 67.9 ± 9.2 | 71.1 ± 8.9 | **<0.001** |
| Age group, years | **40- < 65, %** | 29.8 | 19.7 | **0.012** |
|  | **65 - < 75, %** | 47.5 | 46.3 |  |
|  | **≥ 75, %** | 22.7 | 33.9 |  |
| Male, % | | 33.9 | 42.1 | 0.298 |
| Mean BMI, kg/m^2^ | | 23.7 ± 3.5 | 24.4 ± 3.2 | **0.039** |
| BMI categories, kg/m^2^ | **Underweight, %** | 3.0 | 2.3 | 0.214 |
|  | **Normal, %** | 64.6 | 57.1 |  |
|  | **Overweight, %** | 32.3 | 40.6 |  |
| Office | **SBP, mmHg** | 144.8 ± 16.6 | 139.7 ± 17.1 | **0.002** |
|  | **DBP, mmHg** | 88.4 ± 10.9 | 82.6 ± 10.2 | **<0.001** |
|  | **PR, bpm** | 74.2 ± 10.9 | 74.0 ± 11.0 | 0.851 |
| Morning Home | **SBP, mmHg** | 138.9 ± 13.4 | 135.3 ± 12.5 | **0.006** |
|  | **DBP, mmHg** | 86.2 ± 8.6 | 81.8 ± 9.2 | **<0.001** |
|  | **PR, bpm** | 66.5 ± 9.0 | 65.6 ± 8.3 | 0.246 |
| Evening Home | **SBP, mmHg** | 130.1 ± 11.9 | 127.2 ± 12.3 | **0.015** |
|  | **DBP, mmHg** | 79.5 ± 8.6 | 75.2 ± 8.7 | **<0.001** |
|  | **PR, bpm** | 70.7 ± 8.9 | 69.9 ± 9.2 | 0.418 |
| Living alone, % | | 9.1 | 17.0 | **0.018** |
| Alcohol consumption, % | | 56.1 | 46.8 | 0.059 |
| Educational level ≤ 12years, % | | 44.9 | 55.1 | 0.147 |
| Current or ex-smoker, % | | 40.4 | 39.4 | 0.843 |
| Diabetes, % | | 13.1 | 22.9 | **0.010** |
| Dyslipidemia, % | | 52.5 | 71.6 | **<0.001** |
| History of CVD, % | | 2.0 | 11.5 | **<0.001** |
| Chronic Kidney Disease, % | | 15.7 | 29.4 | **0.007** |
| Antithrombotic drug, % | | 3.5 | 16.1 | **<0.001** |

Data are presented as mean ± standard deviation or number (percentage). Group differences were assessed using Student’s *t*-test for continuous variables and chi-squared test or Fisher’s exact test, as appropriate, for categorical variables. P-values < 0.05 were considered statistically significant.
Abbreviations: *BP*, Blood Pressure; *SBP*, Systolic Blood Pressure; *DBP*, Diastolic Blood Pressure; *PR*, Pulse Rate; *BMI*, Body Mass Index; *CVD*, Cardiovascular disease, BMI categories were defined as underweight (<18.5 kg/m²), normal weight (18.5–24.9 kg/m²), and overweight (≥25.0 kg/m²).

Supplementary Figure 1 Prevalence of white-coat and masked hypertension

Data are presented as percentages within each group: overall hypertensive participants (N = 416), untreated hypertension (N = 198), and treated hypertension (N = 218).
White-coat hypertension: Defined as office BP ≥140/90 mmHg and morning home BP <135/85 mmHg.
Masked hypertension: Defined as office BP <140/90 mmHg and morning home BP ≥135/85 mmHg.
Abbreviations: *BP*, Blood Pressure

Supplementary Figure 2 Distribution of anti-hypertensive medication regimens among treated hypertensive participants (N = 218)


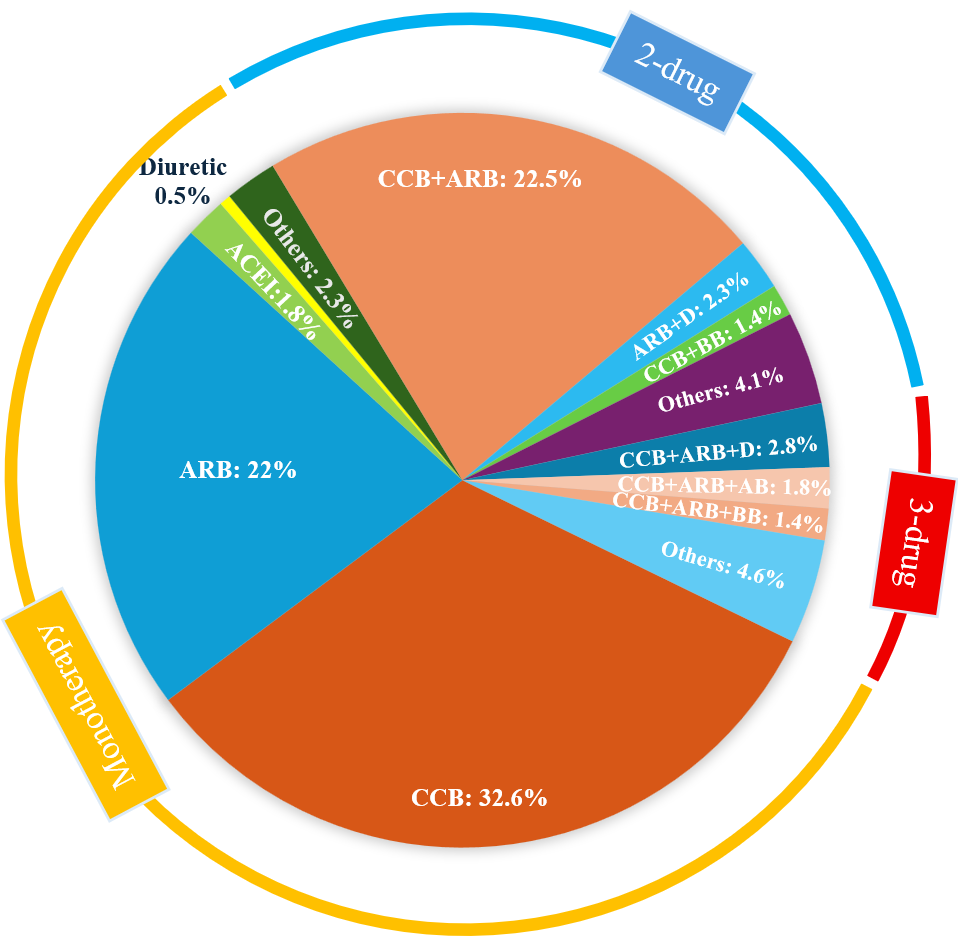


The figure shows the proportion of patients receiving monotherapy, two-drug combinations, and three-drug or more combinations, as well as the specific anti-hypertensive drug classes and combinations used.
Percentages represent the distribution of anti-hypertensive treatment regimens among treated participants, with proportions calculated across all treatment regimens rather than individual drug classes.
Abbreviations: *CCB*, calcium channel blocker; *ARB*, angiotensin II receptor blocker; *ACEI*, angiotensin-converting enzyme inhibitor; *D*, diuretic; *BB*, beta-blocker; *AB*, alpha-blocker.
